# A large-scale species level dated angiosperm phylogeny for evolutionary and ecological analyses

**DOI:** 10.3897/BDJ.8.e39677

**Published:** 2020-01-21

**Authors:** Steven B. Janssens, Thomas L.P. Couvreur, Arne Mertens, Gilles Dauby, Leo-Paul M. J. Dagallier, Samuel Vanden Abeele, Filip Vandelook, Maurizio Mascarello, Hans Beeckman, Marc Sosef, Vincent Droissart, Michelle van der Bank, Olivier Maurin, William Hawthorne, Cicely Marshall, Maxime Réjou-Méchain, Denis Beina, Fidele Baya, Vincent Merckx, Brecht Verstraete, Olivier Hardy

**Affiliations:** 1 Botanic Garden Meise, Meise, Belgium Botanic Garden Meise Meise Belgium; 2 Laboratory for Plant Conservation and Population Biology, KULeuven, Leuven, Belgium Laboratory for Plant Conservation and Population Biology, KULeuven Leuven Belgium; 3 DIADE, IRD, Univ. Montpellier, Montpellier, France DIADE, IRD, Univ. Montpellier Montpellier France; 4 AMAP Lab, IRD, CIRAD, CNRS, INRA, Univ Montpellier, Montpellier, France AMAP Lab, IRD, CIRAD, CNRS, INRA, Univ Montpellier Montpellier France; 5 RMCA, Tervuren, Belgium RMCA Tervuren Belgium; 6 University of Johannesburg, Johannesburg, South Africa University of Johannesburg Johannesburg South Africa; 7 Royal Botanic Gardens, Kew, United Kingdom Royal Botanic Gardens Kew United Kingdom; 8 Department of Plant Sciences, University of Oxford, Oxford, United Kingdom Department of Plant Sciences, University of Oxford Oxford United Kingdom; 9 Department of Plant Sciences, University of Cambridge, Cambridge, United Kingdom Department of Plant Sciences, University of Cambridge Cambridge United Kingdom; 10 Université de Bangui – Cerphameta, Bangui, Central African Republic Université de Bangui – Cerphameta Bangui Central African Republic; 11 Ministère des Eaux, Forêts, Chasse et Pêche, Bangui, Central African Republic Ministère des Eaux, Forêts, Chasse et Pêche Bangui Central African Republic; 12 Department of Evolutionary and Population Biology, University of Amsterdam, Amsterdam, Netherlands Department of Evolutionary and Population Biology, University of Amsterdam Amsterdam Netherlands; 13 Understanding Evolution Group, Naturalis Biodiversity Center, Leiden, Netherlands Understanding Evolution Group, Naturalis Biodiversity Center Leiden Netherlands; 14 Natural History Museum, University of Oslo, Oslo, Norway Natural History Museum, University of Oslo Oslo Norway; 15 Universite Libre de Bruxelles, Brussels, Belgium Universite Libre de Bruxelles Brussels Belgium

**Keywords:** phylogeny, angiosperms, large-scale dating analyses, evolution, ecology

## Abstract

Phylogenies are a central and indispensable tool for evolutionary and ecological research. Even though most angiosperm families are well investigated from a phylogenetic point of view, there are far less possibilities to carry out large-scale meta-analyses at order level or higher. Here, we reconstructed a large-scale dated phylogeny including nearly 1/8th of all angiosperm species, based on two plastid barcoding genes, *matK* (incl. *trnK*) and *rbcL.* Novel sequences were generated for several species, while the rest of the data were mined from GenBank. The resulting tree was dated using 56 angiosperm fossils as calibration points. The resulting megaphylogeny is one of the largest dated phylogenetic tree of angiosperms yet, consisting of 36,101 sampled species, representing 8,399 genera, 426 families and all orders. This novel framework will be useful for investigating different broad scale research questions in ecological and evolutionary biology.

## Introduction

During the past two decades, awareness has grown that ecological and evolutionary studies benefit from incorporating phylogenetic information (Wanntorp et al. 1990, Webb et al. 2002). In some ecological disciplines, it has even become almost unimaginable that a spatiotemporal context is not considered when specific hypotheses are tested. For example, in the fields of community ecology, trait-based ecology and macroecology, macroevolutionary and historical biogeography research hypotheses cannot be properly tested without the incorporation of a phylogenetic framework (e.g. Graham and Fine 2008, Hardy 2008, Kissling 2017, Vandelook et al. 2012, Vandelook et al. 2018, Couvreur et al. 2011, Janssens et al. 2009,Janssens et al. 2016). Likewise, phylogenetic diversity is considered an important element in conservation biology and related biodiversity assessment studies (Chave et al. 2007). Even though the importance of phylogenetics in ecology and evolution is recognised, it remains somehow strenuous to combine ecological research with evolutionary biology and integrate it in a phylogenetic scenario. This discrepancy is sometimes caused by a lack of awareness and knowledge about the other disciplines, whereby researchers could be reluctant to reach out to such expertise and combine their results into new disciplines. Additionally, differences in methodologies and techniques applied by ecologists and evolutionary biologists can sometimes cause a certain hesitation to go for a complementary approach with blending disciplines. In addition, there is a nearly continuous development of new insights and techniques in the fields of ecology and evolution (e.g. Bouckaert et al. 2019, Revell et al. 2008, Revell 2012, Suchard et al. 2018), making it rather challenging to keep up to date with the latest novelties. Furthermore, not all organisms investigated from an ecological perspective are present in molecular databases, which make it difficult to construct a perfectly matching phylogenetic hypothesis for further analysis. For scientists who focus on resolving specific evolutionary or ecological queries, building a phylogenetic framework from novel gene sequence data is often a heavy burden as it takes a lot of time, money and effort, even apart from the specific expertise needed. The construction of a purpose-built phylogeny can be considered as rather costly and labour-intensive and requires more elaborate expertise on novel techniques than when sequences are merely mined from GenBank in order to make a tree, based on already existing sequences. Whereas the former strategy allows the user to make a tailor-made phylogeny that can be used for further ecological or evolutionary purposes, the latter is less proficient, as one can only use the sequences that are available in genetic databases. Nevertheless, in the case of large-scale meta-analyses, it becomes almost impossible to obtain sequence data from all species investigated. When there is a need to examine evolutionary and ecological trends in an historical context, a large-scale phylogenetic hypothesis, that is optimised in a spatiotemporal context, provides an optimal solution.

There is currently an ongoing quest to optimise the methodology for constructing large-scale mega-phylogenies that can be used for further ecological and evolutionary studies. This is done by either mining and analysing publicly available DNA sequences (Zanne et al. 2014), amalgamating published phylograms (Hinchliff et al. 2015) or the combination of both (Smith and Brown 2018). For example, Zanne et al. (2014) constructed their own large supermatrix-based phylogeny that was used to gain more insights into the evolution of cold-tolerant angiosperm lineages. However, the study of Qian and Jin (2016) showed that the phylogeny of Zanne et al. (2014) contained several taxonomic errors. The approaches of Smith and Brown (2018) and Hinchliff et al. (2015) also do not always provide the most optimal phylogenetic framework for further analyses as both studies use a (partially) synthetic approach, based on already published phylograms that can putatively contain inconsistencies in their estimated node ages. The main goal of the present study is, therefore, to provide a large-scale dated phylogeny - encompassing nearly 1/8th of all angiosperms - that can be used for further ecological and evolutionary analyses. In order to construct this angiosperm phylogeny, a comprehensive approach was applied in which sequence data were both mined and generated, subsequently aligned, phylogenetically analysed and dated using over 50 fossil calibration points. With the applied methodology, we aimed to create sufficient overlap in molecular markers without having too much missing sequence data in the datamatrix. In addition, phylogenetic analyses, as well as the age estimation assessment, were performed as a single analysis on the whole datamatrix in order to create a dated angiosperm mega-phylogeny that is characterised by a low degree of synthesis.

## Material and methods

### Marker choice

In 2009, the Consortium for the Barcode of Life working group (CBOL) advised sequencing of the two plastid markers *matK* (incl. *trnK*) and *rbcL* for identifying plant species, resulting in a massive amount of data available on GenBank. *rbcL* is a conservative locus with low level of variation across flowering plants and therefore useful for reconstructing higher level divergence. In contrast, *matK* contains rapidly evolving regions that are useful for studying interspecific divergence (Hilu et al. 2003, Kress et al. 2005). Thus, the combination of *matK* (incl. *trnK*) and *rbcL* has the advantage of combining different evolutionary rates, making it possible to infer relationships at different taxonomic levels. In addition, we sampled only *matK* (incl. *trnK*) and *rbcL* markers in order to reduce missing data to a minimum, as this impacts the phylogenetic inference between species. These supermatrix approaches - which generally contain a substantial amount of missing data – can suffer from imbalance in presence/absence for each taxon per locus, resulting in low resolution and support or even wrongly inferred relationships (Sanderson and Shaffer 2002, Roure et al. 2013).

### Taxon sampling

We extracted angiosperm sequence data of *rbcL* and *matK* (incl. *trnK*) from GenBank (15 February 2015) using the ‘NCBI Nucleotide extraction’ tool in Geneious v11.0 (Auckland, New Zealand). Five gymnosperm genera were chosen as outgroup (Suppl. material 1). This large dataset was supplemented with 468 specimens of African tree species obtained via multiple barcoding projects (available at the Barcode of Life Data Systems (BOLD)), as well as via additional lab work (see paragraph on molecular protocols below). In total, 820 newly obtained sequences are submitted to GenBank (Suppl. material 1).

### Molecular protocols

A modified CTAB protocol was used for total genomic DNA isolation (Tel-Zur et al. 1999). Secondary metabolites were removed by washing ground leaf material with extraction buffer (100 mM Tris pH 8, 5mM EDTA pH 8, 0.35 M sorbitol). After the addition of 575 µl CTAB lysis buffer with addition of 3% PVP-40, the samples were incubated for 1.5 hours (60°C). Chloroform-isoamylalcohol (24/1 v/v) extraction was done twice, followed by an ethanol-salt precipitation (absolute ethanol, sodium acetate 3 M). After centrifugation, the pellet was washed twice (70% ethanol), air-dried and dissolved in 100 µl TE buffer (10 mM Tris pH 8, 1 mM EDTA pH 8).

Amplification reactions of *matK* (incl. *trnK*) and *rbcL* were carried out with a 25 μl reaction mix containing 1 µl DNA, 2 x 1 µl oligonucleotide primer (100 ng/µl), 2.5 µl of 10 mM dNTPs, 2.5 µl Taq Buffer, 0.2 µl KAPA Taq DNA polymerase and 16.8 µl MilliQ water. Reactions commenced with a 3 minute heating at 95°C, followed by 30 cycles consisting of 95°C denaturation for 30 s, primer annealing for 60 s and extension at 72°C for 60 s. Reactions ended with a 3 minute incubation at 72°C. Annealing temperatures for *matK* (incl. *trnK*) and *rbcL* were set at 50°C and 55°C, respectively. Primers designed by Kim J. (unpublished) were used to sequence *matK* (incl. *trnK*), whereas *rbcL* primers were adopted from Fay et al. (1997) and Little and Barrington (2003). PCR products were cleaned using an ExoSap purification protocol. Purified amplification products were sequenced by the Macrogen sequencing facilities (Macrogen, Seoul, South Korea). Raw sequences were assembled using Geneious v11.0 (Biomatters, New Zealand).

### Sequence alignment and phylogenetic analyses

We are aware that the publicly available database, GenBank, contains a large amount of erroneous data (Ashelford et al. 2005, Yao et al. 2004, Shen et al. 2013). Retrieving the sequence data was, therefore, subjected to a quality control procedure. All downloaded sequences were blasted (Megablast option) against the GenBank database, thereby discarding all sequences with anomalies against their original identification. Minimum similarity in BLAST was set at 0.0005, whereas word size (W) was reduced to 8 for greater sensitivity of the local pairwise alignment and the maximum hits was set at 250. A single sequence of each fragment was retained for each taxon name or non-canonical NCBI taxon identifier given in GenBank. In the case where multiple accessions per species were available on GenBank, we chose the accession with the highest sequence length, the best quality and the highest sequence similarity compared to the other accessions of the same species in the GenBank database. Additionally, sequences with multiple ambiguities were discarded, as well as sequences with similar taxon names, but different nucleotide sequences. In addition, sequences with erroneous taxonomic names (checked in R using the “Taxize” and “Taxonstand” packages (R Development Core Team 2009, Cayuela et al. 2012, Chamberlain et al. 2016)) were removed from further analyses. Importantly, Taxize uses the Taxonomic Name Resolution Service (TNRS; Boyle et al. 2013) function to match taxonomic names, whereas Taxonstand is linked with ‘The Plant List’ database. As such, we also checked the validity of the taxonomic names in our dataset using both databases. Only those taxa which had names that were considered valid for both databases were kept for further analyses.

For sequence fragments that are protein-encoded, comparison of amino acid (AA) sequences, based on the associated triplet codons between taxa, was applied. As a result, taxa with a sudden shift in AA or frame shift were discarded from the dataset.

Alignment was carried out in multiple stages. Due to our large angiosperm-wide dataset, an initial alignment (automatically and manually) was conducted for each order included in the dataset. Subsequently, the different alignments were combined using the Profile alignment algorithm (Geneious v11.0, Auckland, New Zealand). The initial automatic alignment was conducted with MAFFT (Katoh et al. 2002) using an E-INS-i algorithm, a 100PAM/k = 2 scoring matrix, a gap open penalty of 1.3, and an offset value of 0.123. Manual fine-tuning of the aligned dataset was performed in Geneious v11.0 (Auckland, New Zealand). During the manual alignment of the different datasets, we carefully assessed the homology of every nucleotide at each position in the alignment (Phillips et al. 2009). The large amount of angiosperm taxa included in the analyses often provided a good view on the evolution of the nucleotides at certain positions, in which some taxa functioned as transition lineages between differing nucleotides and their exact position in the alignment. The importance of a well-designed homology assessment for a complex sequence dataset has been proven successful here for the phylogenetic inference of the angiosperms.

The best-fit nucleotide substitution model for both *rbcL* and *matK* (incl. *trnK*) was selected using jModelTest 2.1.4. (Posada 2008) out of 88 possible models under the Akaike Information Criterion (AIC). The GTR+G model was determined as the best substitution model for each locus and, as such, both markers were jointly analysed under this model. Maximum Likelihood (ML) tree inference was conducted using the Randomized Axelerated Maximum Likelihood (RAxML) software version 7.4.2 (Stamatakis 2006) under the general time-reversible (GTR) substitution model with gamma rate heterogeneity and lewis correction. Although the phylogeny, based on the plastid dataset, generated relationships that corresponded well with currently known angiosperm phylogenies (e.g. Wikström et al. 2001, Soltis et al. 2002, Moore et al. 2007, Magallón and Castillo 2009, Magallón 2014, Magallón et al. 2015, Bell et al. 2005, Bell et al. 2010), we decided to use a constraint (Suppl. material 2) in order to make sure that possible unrecognised mismatches for certain puzzling lineages were significantly reduced. The constraint tree follows the phylogenetic framework of APG4 (Angiosperm Phylogeny Group 2016) at order level. At the lower phylogenetic level, families were only constrained as polytomy in their specific angiosperm order. Genera and species were not constrained.

Support values for the large angiosperm dataset were obtained via the rapid bootstrapping algorithm as implemented in RAxML 7.4.2 (Stamatakis 2006), examining 1000 pseudo-replicates under the same parameters as for the heuristic ML analyses. Bootstrap values were visualised using the Consensus Tree Builder algorithm as implemented in Geneious v11.0.

### Divergence time analysis

Evaluation of fossil calibration points was carried out following the specimen-based approach for assessing paleontological data by Parham et al. (2012). As such, 56 angiosperm fossils were used as calibration points in our molecular dating analysis. Detailed information about the fossils, including (1) citation of museum specimens, (2) locality and stratigraphy of fossils, (3) referenced stratigraphic age and (4) crown/stem node position is provided in Table 1. Fossils are placed at both early and recently diversified lineages within the angiosperms. Due to the large size of the dataset, we applied the penalised likelihood algorithm as implemented in treePL (Smith and O'Meara 2012), which utilises hard minimum and maximum age constraints. In order to estimate these hard minimum and maximum age constraints, we calculated the log normal distribution of each fossil calibration point using BEAUti v.1.10 (Suchard et al. 2018). Maximum age constraints for each fossil correspond to the 95.0% upper boundary of the computed log normal distribution, in which the offset equals the age of the fossil calibration point, the mean is set at 1.0 and the standard deviation at 1.0. This methodology resulted in a minimum 15 million year broad interval for each angiosperm calibration point (Table 1). Due to recently published studies in which both old and young age estimates were retrieved for the crown node of the angiosperms (e.g. Bell et al. 2005, Bell et al. 2010, Magallón et al. 2015, Magallón 2014, Magallón and Castillo 2009, Moore et al. 2007, Smith et al. 2010, Wikström et al. 2001, Soltis et al. 2002), we opted to set the hard maximum and minimum calibration of the angiosperms at 220 and 180 million years, respectively. As for the overall calibration, we followed the strategy of Smith et al. (2010), in which all fossils were considered as a minimum-age constraint. Smith et al. (2010) applied this approach since earlier studies on angiosperm evolution had treated tricolpate fossil pollen as maximum-age constraint, thereby maybe artificially pushing the root age of the angiosperms towards more recent times (e.g. Soltis et al. 2002, Magallón et al. 2015, Magallón 2014, Magallón and Castillo 2009, Moore et al. 2007, Bell et al. 2010, Bell et al. 2005).

The molecular clock hypothesis was tested using a chi^2^ likelihood ratio test (Felsenstein 1988) and demonstrated that the substitution rates in the combined dataset are not clock-like (P < 0.001 for all markers). The most optimal maximum likelihood tree obtained via RAxML was used as input for the penalised likelihood dating analysis in treePL (Smith and O'Meara 2012). Due to the large size dataset, treePL was preferred over other age estimation software packages such as BEAST 1.10 (Suchard et al. 2018), BEAST 2.5 (Bouckaert et al. 2019) or MrBayes 3.2 (Ronquist et al. 2012). The best-fit smoothing parameter of 0.0033 was specified empirically using an adaptation of the cross-validation test as implemented in treePL (Sanderson 2003, Smith and O'Meara 2012). An adapted methodology was set up as the original tree of over 35,000 taxa was too large for correctly calculating the best-fit smoothing parameter. In order to accurately carry out the cross-validation test, 500 replicates were made of the original dataset in which 90% of the original species were randomly pruned. Each of the replicates was then subjected to a cross-validation test under the following parameters: cvstart = 10; cvstop = 0.0001; cvmultstep = 0.9; randomcv. The best-fit smoothing parameter was selected as the variable with the highest proportion (0.0033; 12%), with the second best-fit smoothing parameter being situated at 0.0036 (11%). Smoothing parameters calculated per replicate followed a normal distribution with its optimum around 0.0033 and 0.0036 (Suppl. material 3). This strategy of calculating the smoothing parameter of very large datasets seemed effective and robust for estimating node ages of our angiosperm phylogeny using treePL. Furthermore, since there is a large amount of rate heterogeneity amongst angiosperm lineages that could likely infringe the treePL model, it is considered that a low smoothing parameter will provide a more robust analysis. So, by applying a lower penalty, potential issues that could be caused by strongly contrasting evolutionary rates within distant angiosperm clades will putatively be avoided (Stephen Smith, pers. comm.). In order to generate 95% confidence intervals for the dated nodes, we generated 1,000 bootstrap pseudo-replicates using the ML topology of the earlier heuristic analysis as constraint. Each ML bootstrap tree was then individually dated using treePL under the same parameters as for the single age estimation analysis, described above. Subsequently, the 1,000 dated bootstrap trees were imported into TreeAnnotator v1.10 in order to calculate and visualise the 95% confidence intervals for each node (Suchard et al. 2018).

## Results and Discussion

The final aligned data matrix consists of 36,101 angiosperm species. *matK* (incl. *trnK*) sequences were mined for 31,391 species (87%), whereas *rbcL* sequences were obtained for 26,811 (74%) species (Suppl. material 1). The sequence dataset has an aligned length of 4,968 basepairs (bp) of which 4,285 (86%) belong to *matK* (incl. *trnK*) and 683 (14%) to *rbcL*. Within *rbcL*, all characters were variable (100%), whereas for *matK* (incl. *trnK*) 3,921 characters (91.5%) were variable. Support value analyses indicate that approximately 26% of the branches have a bootstrap value > 75 (Suppl. material 4Suppl. material 3). Based on the different studies that estimated the total number of flowering plants currently described (between 260,000 and 450,000 species) (Crane et al. 1995, Christenhusz and Byng 2016, Cronquist 1981, Lupia et al. 1999, Pimm and Joppa 2015, Prance et al. 2000, Thorne 2002), the presented phylogeny represents between 14% and 8% of the known flowering plants, respectively. In addition, the phylogenetic tree contains 54.6% (8,399) of all currently accepted angiosperm genera and 94.5% (426) of all families of flowering plants are included, as well as all currently known angiosperm orders. As such, the current angiosperm tree can be regarded as the largest dated angiosperm phylogenetic framework that is generated by combining genuine sequence data and fossil calibration points and will be useful for large-scale ecological and biogeographical studies. Compared to the species-level-based tree of Zanne et al. (2014) and its updated version by Qian and Jin (2016), the current phylogeny is larger in size, containing more species (+4,797 species) and genera (+468). However, the phylogeny of Zanne et al. (2014) included more families and an equal number of orders. Additionally, Zanne et al. (2014)'s updated phylogeny (Qian and Jin 2016) also included 1,190 taxa of bryophytes, pteridophytes and gymnosperms, whereas the current phylogeny only contains 5 outgroup gymnosperm species. As a result, when comparing the differences in species number between both angiosperm mega-phylogenies, the current tree contains nearly 20% more flowering plant lineages (+5,987 species).

**Figure 1.**
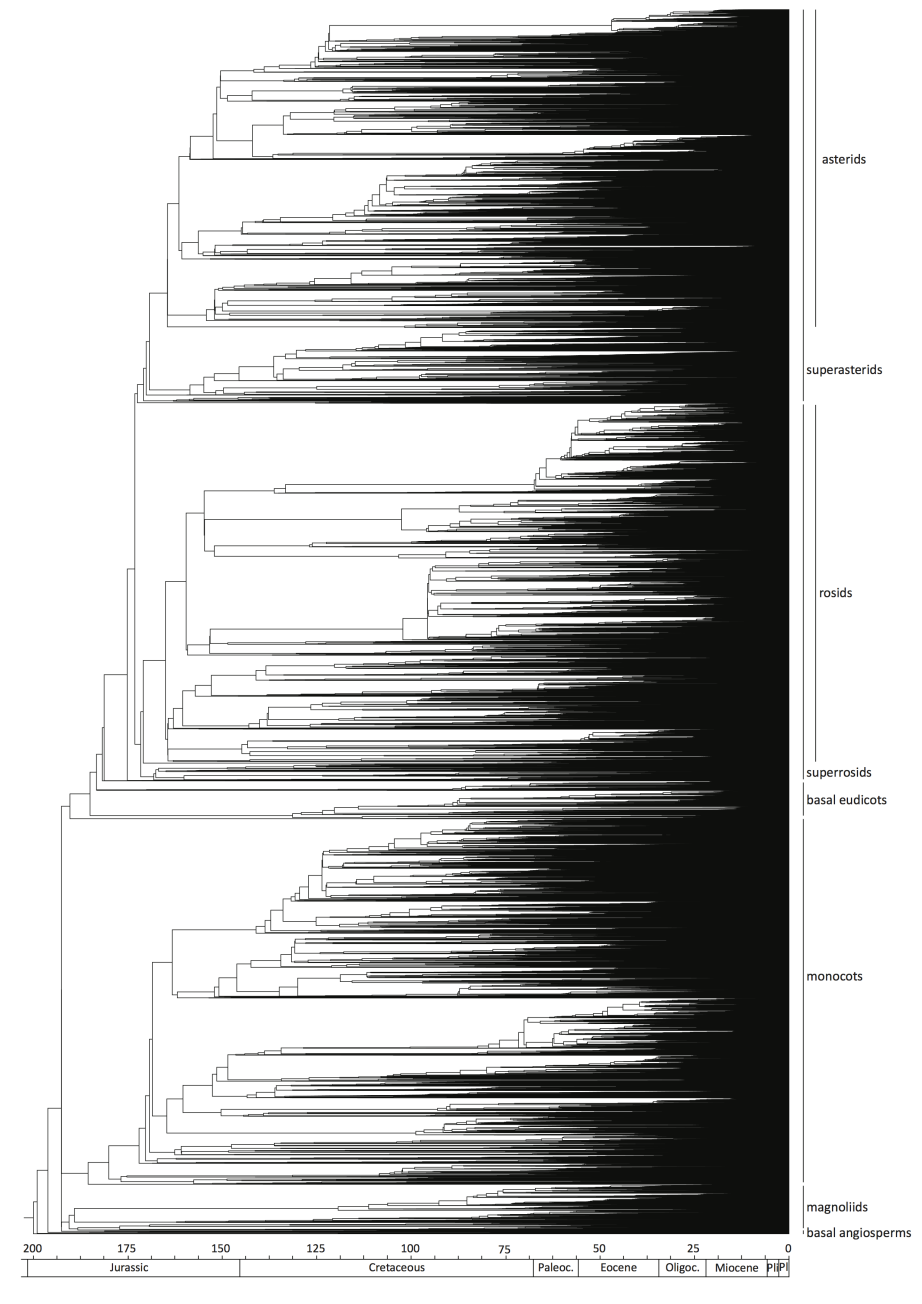
Maximum Likelihood-based angiosperm phylogram based on the combined *rbcL* and *matK* (incl. *trnK*) dataset.

Age estimation of the large-scale angiosperm tree resulted in a dated phylogeny (Fig. 1; Suppl. material 5) that largely corresponds to the different recent angiosperm-wide dating analyses (e.g.Bell et al. 2010, Magallón et al. 2015, Smith et al. 2010, Wikström et al. 2001, Zanne et al. 2014). Even though small dissimilarities are present concerning the age of the most early diversified angiosperm lineages (see Table 1), the overall age of the different families corresponds rather well to what is known from these other studies. Differences in stem node age of large clades such as superasterids, superrosids, eudicots, monocots or magnoliids are probably due to the use of a slightly different and larger set of fossil calibration points, as well as not using tricolpate fossil pollen as maximum-age for eudicots. Compared to the angiosperm phylogeny of Zanne et al. (2014), where time-scaling was carried out with 39 fossil calibrations, the current tree contains 56 fossils in total. Although some fossils are the same between both Zanne’s study and ours (e.g. *Pseudosalix handleyi*, *Fraxinus wilcoxiana*, *Spirematospermum chandlerae*), several fossils that have been used to optimise the age estimation of the current megaphylogeny are carefully chosen from other dating analyses (Bell et al. 2010, Magallón et al. 2015, Smith et al. 2010).

Recently, Qian and Jin (2016) developed a novel tool (S.PhyloMaker package as implemented in the R environment) to generate artificially enriched species trees, based on an updated version of the original angiosperm mega-phylogeny of Zanne et al. (2014). According to the study of Qian and Jin (2016), the software package produces phylogenies for every species that one needs to assess in a community ecological environment. S.PhyloMaker grafts species of interest, either as a basal polytomy (regular or Phylomatic/BLADJ approach; Webb et al. 2008), or randomly branched within the existing parental clades that are found in the mega-phylogeny. Likewise, branch lengths or time-calibrated node splits of newly added taxa are also artificially estimated according to their relative position in the original mega-phylogeny. Even though the software package of Qian and Jin (2016) provides a good alternative for the lack of decent sampling of angiosperm taxa in mega-phylogenies for some ecological studies, not all ecological or evolutionary disciplines that are in need of a phylogenetic framework can rely on this methodology, as it is not based on the inclusion of original sequence data. Additionally, the current, more densely sampled phylogenetic framework could be used in the S.Phylomaker system in order to reduce the variance that is related to the random addition of new lineages, as the placement of new taxa can be more precisely carried out due to the presence of more nodes with known heights. The use of only chloroplast data for the construction of this large-scale angiosperm mega-phylogeny has, indeed, some disadvantages as chloroplasts constitute a single, linked locus that is mainly maternally inherited within angiosperms and processes such as hybridisation and subsequent introgression, as well as reticulate evolution and incomplete lineage sorting, are difficult to detect with only data from one genome (Soltis and Soltis 2009, Lee et al. 2011). This, in combination with the fact that only two gene markers were used for phylogeny reconstruction, results in making this phylogeny to be regarded as an angiosperm gene tree rather than a species tree. Despite these putative issues, the large-scale phylogenetic hypothesis, that has been constructed here, has proven to be useful for resolving large-scale evolutionary questions at angiosperm level (e.g. Dagallier et al. in press). To date, it remains a continuous challenge to increase the size of large-scale angiosperm phylogenies with new species and gene markers to create a reliable platform, in which ecological and evolutionary research can be combined with phylogenetics. The current phylogeny is a further step towards an all-encompassing angiosperm phylogeny that can be used to resolve large-scale ecological and evolutionary queries.

## Supplementary Material

7E4B98BD-612D-51CD-B1F3-B7A3B7617711 10.3897/BDJ.8.e39677.suppl1 Supplementary material 1 Supplementary Table Data type: Species list
Brief description: Table S1. Accession numbers of *rbcL* and *matK* (incl. *trnK*) sequences of the species included in the angiosperm phylogeny (including information on genera, family and order). Newly obtained accessions are indicated with an asterisk.
File: oo_329737.xlsx https://binary.pensoft.net/file/329737 Steven Janssens

05804820-2936-5679-8CA6-7EFAA15299E5 10.3897/BDJ.8.e39677.suppl2 Supplementary material 2 Constraint input topology Data type: Constraint topology
Brief description: Constraint input topology for RAxML analyses of all angiosperms analysed in this study (incl. outgroup taxa).
File: oo_362680.tre https://binary.pensoft.net/file/362680 Steven Janssens

7B0807DC-D59F-5817-BD67-B4227D2C7A31 10.3897/BDJ.8.e39677.suppl3 Supplementary material 3 Proportion of smoothing parameters Data type: graph
Brief description: Proportion of smoothing parameters calculated for each of the 500 tree replicates
File: oo_363086.pdf https://binary.pensoft.net/file/363086 Steven Janssens

556B2B61-14D6-5B99-BE55-0D1EC70F1F6D 10.3897/BDJ.8.e39677.suppl4 Supplementary material 4 Angiosperm phylogeny - ML bootstrap values Data type: phylogeny
Brief description: Maximum Likelihood bootstrap consensus tree. Values above the branches indicate bootstrap support. Note that the support values above order level are all artificially set at 100 because of the use of a constraint backbone.
File: oo_329452.tre https://binary.pensoft.net/file/329452 Steven Janssens

8CA97B3B-DFB5-5CBC-B8B1-C9D826C26780 10.3897/BDJ.8.e39677.suppl5 Supplementary material 5 Dated angiosperm phylogram Data type: phylogeny
Brief description: Maximum Likelihood phylogram of 36101 angiosperm species (nexus file). Outgroup included. Blue bars indicate 95% confidence intervals.
File: oo_330891.tre https://binary.pensoft.net/file/330891 Steven Janssens

## Figures and Tables

**Table 1.** List of fossils used as calibration points, including their oldest stratigraphic occurrence, minimum and maximum ages, the calibrated clades and used references. cr.=crown, st.=stem.

**Clade**	**Fossil**	**Reference**	**Period**	**Locality/Formation/Group**	**Min. age**	**Max. age**	**cr. / st.**
Ebenaceae	*Austrodiospyros cryptostoma* Basinger et Christophel	Basinger and Christophel 1985	Late Eocene	Anglesea formation (Victoria, Australia)	37.8	54.62	cr.
Apocynaceae	*Apocynophyllum helveticum* Heer	Wilde 1989	Middle Eocene	Messel formation (Darmstadt, Germany)	47.8	64.62	cr.
Cornaceae	*Hironoia fusiformis* Takahashi, Crane et Manchester	Takahashi et al. 2002	Early Conacian	Ashizawa formation, Futuba group (North-eastern Honshu, Japan)	89.8	106.6	cr.
Dipelta	*Dipelta europaea* Reid et Chandler	Reid and Chandler 1926	Late Eocene-Early Oligocene	Bembridge Flora (UK)	33.9	50.72	st.
Oleaceae	*Fraxinus wilcoxiana* (Berry) Call et Dilcher	Call and Dilcher 1992	Middle Eocene	Claiborne formation (Tennessee, USA)	47.8	64.62	st.
Diervilla	*Diervilla echinata* Piel	Piel 1971	Oligocene	Fraser River system (British Colombia, Canada)	27.8	44.62	st.
Solanaceae (Physalinae)	*Physalis infinemundi* Wilf, Carvahlo, Gandolfo et Cuneo	Wilf et al. 2017	Early Eocene	Laguna del Hunco (Chubut, Patagonia, Argentina)	52.0	68.82	st.
Valeriana	*Valeriana* sp.	Mai 1985	Late Miocene	Europe	11.6	28.42	st.
Emmenopterys	*Emmenopterys* Oliv.	Wehr and Manchester 1996	Middle Eocene	Middle Eocene Republic Flora (Washington, USA)	47.8	64.62	st.
Pelliciera	*Pelliciera rhizophorae* Planch. et Triana	Graham 1977	Middle Eocene	Gatuncillo formation (Panama)	47.8	64.62	st.
Araliaceae	*Acanthopanax gigantocarpus* Knobloch et Mai	Knobloch and Mai 1986	Maastrichtian	Eisleben formation (Germany)	72.1	88.92	st.
Ilex	*Ilex hercynica* Mai	Mai 1987	Early Paleocene	Gonna formation (Sangerhausen, Germany)	66.0	82.82	st.
Actinidiaceae	*Saurauia antiqua* Knobloch et Mai	Knobloch and Mai 1986	Late Santonian	Klikov-Schichtenfolge (Germany)	85.8	102.6	st.
Nymphaeales	*unnamed Nymphaeales*	Friis et al. 2001	Late Aptian-Early Albian	Vale de Agua (Portugal)	112.0	128.8	cr.
Canellales	*Walkeripollis gabonensis* Doyle, Hotton et Ward	Doyle et al. 1990	Late Barremian-Early Aptian	Cocobeach (Gabon)	125.0	141.8	st.
Magnoliaceae	*Archaeanthus linnenbergeri* Dilcher et Crane	Dilcher and Crane 1984	Early Cenomanian	Dakota formation (Kansas, USA)	100.5	117.3	cr.
Magnoliales	*Endressinia brasiliana* Mohr et Bernardes-de-Oliveira	Mohr and Bernardes-De-Oliveira 2004	Aptian-Albian	Crato formation (Brasil)	112.0	128.8	cr.
Lauraceae	*Potomacanthus lobatus* Crane, Friis et Pedersen	Crane et al. 1994	Early and Middle Albian	Puddledock locality (Virginia, USA)	119.0	135.8	cr.
Arecaceae	unnamed palms	Christopher 1979, Daghlian 1981	Conacian-Santonian	Magothy formation (Maryland)	89.8	106.6	cr.
Musella-Ensete	*Ensete oregonense* Manchester et Kress	Manchester and Kress 1993	Middle Eocene	Clarno formation (Oregon, USA)	43.0	59.82	st.
Zingiberaceae	*Zingiberopsis attenuata* Hickey et Peterson	Hickey and Peterson 1978	Middle to late Paleocene	Paskapoo formation (Alberta, Canada)	61.6	78.42	cr.
Zingiberales	*Spirematospermum chandlerae* Friis	Friis 1988	Santonian-Campanian	Neuse River formation (North Carolina, USA)	83.6	100.4	cr.
Araceae	*Mayoa portugallica* Friis, Pedersen et Crane	Friis et al. 2004	Barremanian-Aptian	Almargem formation (Torres Vedras, Portugal)	125.0	141.8	cr.
Restionaceae	unnamed Restionaceae	Jarzen 1978	Maastrichtian	Morgan Creek (Saskatchewan, Canada)	72.1	88.92	st.
Poaceae	unnamed grasses	Jardiné and Magloire 1965	Maastrichtian	Senegal-Ivory Coast	72.1	88.92	cr.
Berberidaceae	*Mahonia* Nutt.	Manchester 1999	Middle Eocene	Green River formation (Colorado-Utah, USA)	47.8	64.62	cr.
Platanaceae	*Platanocarpus brookensis Crane*, Pedersen, Friis et Drinnan	Crane et al. 1993	Early and Middle Albian	Patapsco formation (Virginia, USA)	112.0	128.8	st.
Sabiales	*Insitiocarpus moravicus* Knobloch et Mai	Knobloch and Mai 1986	Early Cenomanian	Peruc-schichten (Czeck Republic)	98.0	114.8	cr.
Iteaceae	*Divisestylus brevistamineus*	Hermsen et al. 2003	Turonian	Raritan formation (New Jersey)	93.9	110.7	cr.
Altingiaceae	*Microaltingia apocarpela*	Zhou et al. 2001	Turonian	Raritan formation (New Jersey)	93.9	110.7	cr.
Tilia	*Tilia vescipites* Nichols et Ott	Nichols and Ott 1978	Middle Paleocene	Wind River basin (Wyoming, USA)	61.6	78.42	cr.
Polygonaceae	*Persicaria* (L.) Mill.	Muller 1981	Paleocene	Europe	66.0	82.82	cr.
Clausena	*Clausena* Burm.f.	Pan 2010	Late Oligocene	Guang River Flora (Ethiopia)	27.36	44.18	cr.
Malpighiales	*Paleoclusia chevalieri* Crepet et Nixon	Crepet and Nixon 1998	Turonian	Raritan formation (New Jersey)	93.5	110.3	cr.
Fagales	* Normapolles *	Batten 1981, Kedves 1989, Pacltova 1966	Late Cenomanian	Europa and USA	94.7	111.5	cr.
Phytolaccaceae	*Coahuilacarpon phytolaccoides* Cevallos-Ferriz, Estrada-Ruiz et Perez-Hernandez	Cevallos-Ferriz et al. 2008	Late Campanian	Cerro del Pueblo formation (Mexico)	72.5	89.32	st.
Juglandaceae	*Cyclocarya brownii* Manchester et Dilcher	Crane et al. 1990	Late Paleocene	Almont and Beicegel Creek (North Dakota, USA)	59.2	76.02	cr.
Rosales	*unnamed Rosidae*	Crepet and Nixon 1996	Turonian	Raritan formation (New Jersey)	93.9	110.7	cr.
Betulaceae	*Endressianthus miraensis* Friis, Pedersen et Schoenenberger	Friis et al. 2003	Campanian-Maastrichtian	Mira (Portugal)	72.1	88.92	cr.
Fagaceae	*Antiquacupula sulcata* Sims, Herendeen et Crane	Sims et al. 1998	Late Santonian	Gaillard formation (Georgia, USA)	85.8	102.6	cr.
Salicaceae	*Pseudosalix handleyi* Boucher, Manchester et Judd	Boucher et al. 2003	Middle Eocene	Green River formation (Colorado-Utah, USA)	53.5	70.32	cr.
Ranunculales	*Leefructus mirus* Sun, Dilcher, Wang et Chen	Sun et al. 2011	Barremanian-Aptian	Yixian formation (China)	125.0	141.8	cr.
Fabaceae	*Fabaceae* sp.	Herendeen et al. 1992	Early Eocene	Buchanan clay pit (Tenessee, USA)	56.0	72.82	cr.
Styracaceae	*Rehderodendron stonei* Vaudois-Mieja	Vaudois-Miéja 1983	Early Eocene	Sabals d'Anjou (France)	56.0	72.82	cr.
Dipterocarpaceae	*Shorea maomingensis* Feng, Kodrul et Jin	Feng et al. 2013	Late Eocene	Huangniuling formation (Maoming Basin, China)	37.8	54.62	cr.
Lamiaceae	*Ajuginucula smithii* Reid et Chandler	Reid and Chandler 1926	Late Eocene-Early Oligocene	Bembridge Flora (UK)	33.9	50.72	cr.
Theaceae s.l.	*Pentapetalum trifasciculandricus* Martinez-Millan, Crepet et Nixon	Martinez-Millan et al. 2009	Turonian	Raritan formation (New Jersey)	93.9	110.7	cr.
Myrsinaceae	*unnamed Myrsinaceae*	Pole 1996	Middle Miocene	Foulden Hills Diatomite (New Zealand)	15.9	32.72	cr.
Myrtaceae	*Tristaniandra alleyi* Wilson et Basinger	Basinger et al. 2007	Middle Eocene	Golden Grove - East Yatala Sand Pit (South Australia)	47.8	64.62	cr.
Lythraceae	*Decodon tiffneyi* Estrada-Ruiz, Calvillo-Canadell et Cevallos-Ferriz	Estrada-Ruiz et al. 2009	Late Campanian	Cerro del Pueblo formation (Mexico)	72.5	89.32	cr.
Ampelocissus s.l.	*Ampelocissus parvisemina* Chen et Manchester	Chen and Manchester 2007	Late Paleocene	Beicegal Creek (North Dakota, USA)	59.2	76.02	cr.
Vitaceae	*Indovitis chitaleyae* Manchester, Kapgate et Wen	Manchester et al. 2013	Maastrichtian	Mahurzari (India)	72.1	88.92	cr.
Rosa	*Rosa germerensis* Edelman	Edelman 1975	Early Eocene	Germer Basin Flora (Idaho, USA)	56.0	72.82	cr.
Prunus	*Prunus wutuensis* Li, Smith, Liu, Awasthi, Yang et Li	Li et al. 2011	Early Eocene	Wutu (China)	56.0	72.82	cr.
Myristicaceae	*Myristicacarpum chandlerae* Manchester, Doyle et Sauquet	Doyle et al. 2008	Early Eocene	London Clay (UK)	56.0	72.82	cr.
