# Supplementary figures and images for: A large-scale species level dated angiosperm phylogeny for evolutionary and ecological analyses
Source: Biodivers Data J. 2020 Jan 21;8:e39677. doi: 10.3897/BDJ.8.e39677 (PMC6987248; doi:10.3897/BDJ.8.e39677)

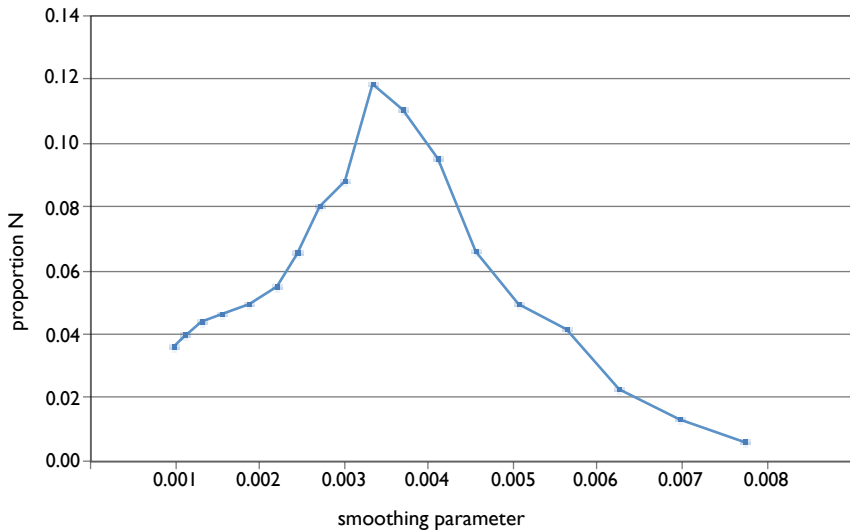

Supplement: Supplementary material 3 — Proportion of smoothing parameters [file bdj-08-e39677-s003.pdf]
